# Hugh Champneys Thurston

**Published:** 1919-12

**Authors:** 


### HUGH CHAMPNEYS THURSTON, C.B., C.M.G.

It is with much regret we have to record the death of Colonel Hugh Champneys Thurston, C.B., C.M.G., Army Medical Service, who died after a very short illness at Torquay on August 17th, 1919, and only a few weeks after he had retired from his arduous duties in the Field.

Hugh Thurston was born on March 14th, 1862. He was the son of the late O. E. Thurston of Kington, near Thornbury, and joined the old Bristol Medical School in the late seventies, where he took a prominent position in the prize lists as well as in the football field. He took the L.R.C.P. Lond. and M.R.C.S. Eng. in 1884, and in the same year he came into residence at the Infirmary as Assistant Resident Officer and Pathologist, showing himself at once to be a practical and energetic man. During the years 1884 and 1885 he paid special attention to Pathology, and the Infirmary Museum still contains many excellent specimens collected and mounted by him.

He was a great favourite in the wards, and was much missed in Bristol when he joined the R.A.M.C. in 1887. He very soon saw active service in Burma with the Wuntho Expedition under Sir Garnet Wolseley in 1891, for which he was awarded the medal and clasp.

In 1896 he married Susie, daughter of the late Charles Steele, M.D., F.R.C.S., of Clifton.

Thurston was promoted Major in 1899, and served in South Africa during the whole of the war, 1899–1902. He was present at the Relief of Kimberley, and took part in the operations at Paardeberg, also in the following actions : Poplar Grove, Zand River, near Johannesburg, Pretoria and Diamond Hill, Reit Vlei and Belfast, and at Colesberg. For these numerous services he was mentioned in despatches, obtained the Queen's Medal with five clasps, and the King's Medal with two clasps, and honoured with the C.M.G.

Hugh Thurston was Deputy Assistant Director-General at the War Office from 1904 to 1908.

In 1908 he presided at the Bristol Medical Dinner, when a very large company attended to greet him.

He was promoted Lieutenant-Colonel in 1911 and Colonel (A.M.S.) in 1915.

He served with much distinction throughout the Great War as A.D.M.S., and acted for a considerable period in 1917 as D.D.M.S. at the important Medical Base at Havre.

During the war he was frequently mentioned in despatches and was honoured with the C.B.

The writer of this notice met him several times in South Africa and in France, when yarns of the B.R.I. and the old School were told. As a junior officer he was always loyal to his Alma Mater, and as a senior officer was always ready to give a cordial welcome to those who hailed from the West Country.

Colonel Thurston leaves a widow, one son and one daughter to lament his loss. He will long be remembered by all who knew him in the years now gone, as a kindly gentleman, an able administrator, and a sound medical man.

